# Genome-wide association study of prevalent and persistent cervical high-risk human papillomavirus (HPV) infection

**DOI:** 10.1186/s12881-020-01156-1

**Published:** 2020-11-23

**Authors:** Sally N. Adebamowo, Adebowale A. Adeyemo, Charles N. Rotimi, Olayinka Olaniyan, Richard Offiong, Clement A. Adebamowo, for the H3Africa ACCME Research Group, Michael Odutola, Eileen O. Dareng, Ayotunde O. Famooto, Ruxton Adebiyi

**Affiliations:** 1 Department of Epidemiology and Public Health, University of Maryland School of Medicine, 660 West Redwood Street, Howard Hall, Room 119, Baltimore, MD 21201 USA; 2 University of Maryland Greenebaum Comprehensive Cancer Center, University of Maryland School of Medicine, Baltimore, MD USA; 3 Center for Research on Genomics and Global Health, National Human Genome Research Institute, National Institutes of Health, Bethesda, MD USA; 4 Department of Obstetrics and Gynecology, National Hospital Abuja, Abuja, Nigeria; 5 Department of Obstetrics and Gynecology, University of Abuja Teaching Hospital, Abuja, Nigeria; 6 Institute of Human Virology, University of Maryland School of Medicine, Baltimore, MD USA; 7 Institute of Human Virology Nigeria, Abuja, Nigeria

## Abstract

**Background:**

Genetic factors may influence the susceptibility to high-risk (hr) human papillomavirus (HPV) infection and persistence. We conducted the first genome-wide association study (GWAS) to identify variants associated with cervical hrHPV infection and persistence.

**Methods:**

Participants were 517 Nigerian women evaluated at baseline and 6 months follow-up visits for HPV. HPV was characterized using SPF_10_/LiPA_25_. hrHPV infection was positive if at least one carcinogenic HPV genotype was detected in a sample provided at the baseline visit and persistent if at least one carcinogenic HPV genotype was detected in each of the samples provided at the baseline and follow-up visits. Genotyping was done using the Illumina Multi-Ethnic Genotyping Array (MEGA) and imputation was done using the African Genome Resources Haplotype Reference Panel. Association analysis was done for hrHPV infection (125 cases/392 controls) and for persistent hrHPV infection (51 cases/355 controls) under additive genetic models adjusted for age, HIV status and the first principal component (PC) of the genotypes.

**Results:**

The mean (±SD) age of the study participants was 38 (±8) years, 48% were HIV negative, 24% were hrHPV positive and 10% had persistent hrHPV infections. No single variant reached genome-wide significance (*p* < 5 X 10^− 8^). The top three variants associated with hrHPV infections were intronic variants clustered in *KLF12* (all OR: 7.06, *p* = 1.43 × 10^− 6^). The top variants associated with cervical hrHPV persistence were in *DAP* (OR: 6.86, *p* = 7.15 × 10^− 8^), *NR5A2* (OR: 3.65, *p* = 2.03 × 10^− 7^) and *MIR365–2* (OR: 7.71, *p* = 2.63 × 10^− 7^) gene regions.

**Conclusions:**

This exploratory GWAS yielded suggestive candidate risk loci for cervical hrHPV infection and persistence. The identified loci have biological annotation and functional data supporting their role in hrHPV infection and persistence. Given our limited sample size, larger discovery and replication studies are warranted to further characterize the reported associations.

**Supplementary Information:**

The online version contains supplementary material available at 10.1186/s12881-020-01156-1.

## Background

Human papillomavirus (HPV) is a highly conserved double-stranded DNA virus that has coevolved with human populations for millennia [1]. Over 150 types of HPV have been identified and about 40 types primarily infect stratified cutaneous or mucosal epithelia [2]. HPV infections are among the most common sexually transmitted infections globally [3]. While most infections are cleared naturally by the host’s immune system in ~ 2 years, the infection persists in about 10% of those affected [4]. Persistent infection by high-risk (hr) HPV is a risk factor for many epithelial cancers including head and neck, anal and cervical cancers. Susceptibility to cervical hrHPV infection, its persistence and progression to neoplastic disease are determined by epidemiologic and genetic factors. Many epidemiologic risk factors for cervical hrHPV infection including oral contraceptives, cigarette smoking, multiple sexual partners and co-infection with HIV are well documented [5–8], however little is known about the genetic risk factors.

Wang *et. al* evaluated a panel of 7140 candidate single nucleotide polymorphisms (SNP) in 305 candidate genes/regions selected based on a priori hypotheses of their association with HPV infection and cervical cancer, within the population-based Guanacaste cohort in Costa Rica. They reported that SNPs in Deoxyuridine Triphosphatase (*DUT*), General Transcription Factor IIH Subunit 4 (*GTF2H4*), 2′-5′-Oligoadenylate Synthetase 3 (*OAS3*) and Sulfatase 1 (*SULF1*) gene regions were associated with HPV persistence, while SNPs in the Transmembrane Channel Like (*TMC*) 6 and *TMC8* gene regions were associated with progression to cervical intraepithelial neoplasia (CIN) 3 and cervical cancer [9]. In a subsequent study in the same cohort, the investigators examined 18,310 SNPs in 1113 genes and reported that SNPs in *PRDX3* and *RPS19* were associated with HPV persistence and progression from persistent HPV infection to CIN3+ [10]. We examined the association between the aforementioned SNPs and prevalent hrHPV infection in African women, and successfully replicated *RPS19*:rs2305809 and *TYMS*:rs2342700 [11].

While the previous candidate gene studies have provided insight into the genetic risk of HPV infection and persistence, agnostic approaches such as genome-wide association studies (GWAS), which interrogate the entire genome would be more useful to uncover novel susceptibility loci for cervical hrHPV infections. A GWAS of cervical hrHPV infection, can also identify novel biomarkers and potential therapeutic targets in cervical cancer, however, none have been conducted to date. We therefore conducted this GWAS of cervical hrHPV infections and tested previously reported associations between genes/regions and prevalent and persistent cervical hrHPV infections.

## Methods

### Study population

We studied 544 women participating in a cohort study of cervical HPV infection and cervical cancer at National Hospital, Abuja and University of Abuja Teaching Hospital, Nigeria, and enrolled between 2012 and 2014, as previously described [5, 12–14]. All the study participants were 18 years of age or older, had a history of vaginal sexual intercourse, were not currently pregnant and had no history of hysterectomy. We collected data on socio-demographic characteristics, sexual and reproductive history, and confirmed participants’ HIV status from hospital medical records at study entry. Participants were asked to return for follow-up visits after 6 months, at which time, the history, physical examinations and sample collections were repeated. We collected venous blood samples and performed pelvic examinations on all the study participants at each study visit. Elution swab system (Copan, Italy) was used to collect exfoliated cervical cells, which were inserted in 1 ml Amies’ transport media (Copan).

### HPV detection by SPF_10_/LiPA_25_

We extracted DNA from the cervical exfoliated cells as previously described [11]. Samples were tested for HPV DNA by hybridization of SPF_10_ amplimers to a mixture of general HPV probes recognizing a broad range of high-risk, low-risk, and possible hrHPV genotypes in a microtiter plate format, as described previously [15]. All samples determined to be HPV DNA positive by SPF_10_ DNA Enzyme Immunoassay (DEIA) were genotyped using the LiPA_25_ version 1. The LiPA_25_ assay provides type-specific information for 25 different HPV genotypes simultaneously and identifies infection by one or more of 13 hrHPV genotypes: 16, 18, 31, 33, 35, 39, 45, 51, 52, 56, 58, 59, and 68 [16, 17]. However, as this assay does not differentiate between HPV 68 and 73, we defined this HPV genotype (i.e. HPV68/73) as low-risk. We defined hrHPV infection as prevalent if at least one hrHPV genotype was detected in the baseline sample and persistent if at least one hrHPV genotype was detected in samples provided at both the baseline and follow-up visits. We defined persistently negative as absence of hrHPV genotype in the baseline and follow-up visit samples.

### Genotyping and imputation

Samples from the study participants were genotyped using the Illumina Multi-Ethnic Global Array (MEGA) which has ~ 1.7 million markers. Sample-level genotype call rate was at least 0.95 for all the study participants. We filtered out from the genotyped dataset SNPs that did not meet the following criteria: autosomal SNPs (*n* = 78,713), variant missingness < 0.05 (*n* = 96,410), Hardy-Weinberg equilibrium (HWE) *p* > 1 × 10–6 (*n* = 7692) and minor allele frequency (MAF) > = 0.01 (*n* = 564,791). The resulting 958,363 SNPs that passed these quality control filters had a SNP success rate of 0.9985 and were used as the basis for imputation.

Imputation was performed using the Sanger Imputation Service (https://imputation.sanger.ac.uk/) [18]. Pre-phasing was done with the Eagle2 algorithm [19] and imputation was done with positional Burrows-Wheeler transform (PBWT) [20]. The reference panel used was the African Genome Resources Haplotype Reference Panel, an African genome imputation reference panel based on 9912 haplotypes (4956 samples) which includes all African and non-African 1000 Genomes Phase 3 populations and additional African genomes from Uganda, Ethiopia, Egypt, Namibia and South Africa (including 2298 African samples with whole genome sequence data from the African Genome Variation Project (AGVP) [21] and the Uganda 2000 Genomes Project (UG2G) [22]. The IMPUTE2 INFO score was used as a quality metric to evaluate the uncertainty in genotype imputation. Imputation yielded a total number of ~ 104 million markers. We filtered the resulting imputation dataset for variants with info score ≥ 0.3 and MAF ≥ 0.01, with a final set of ~ 18 million SNPs which was used for association analysis.

### Statistical analysis

From the original set of 544 women, we excluded 27 women from the baseline analyses because of incomplete data (5 missing HPV, 22 missing both HPV and HIV results), leaving only 517 women in the baseline analyses. Of the 517 women, we excluded those who did not return for the follow-up visit (*n* = 9), those with missing HPV results (*n* = 35) and included the remaining 473 women in the analyses for persistent hrHPV infections. For the prevalent hrHPV analysis, we compared 125 women with cervical hrHPV infections (cases) to 392 women without cervical hrHPV infections at baseline (controls). For the persistent hrHPV analysis, we compared 51 women with hrHPV infection at both the baseline and follow-up visits (cases) to 355 women without hrHPV infections at either the baseline or follow-up visits (controls). Using LD-pruned SNP genotype data available on the same women, we computed principal components based on the variance-standardized relationship matrix with PLINK 1.9 [23, 24] using the parameters *“--indep 50 5 2*” , namely with a window size of 50 SNPs, 5 SNPs to shift the window at each step and a variance inflation factor of 2. We found that the first principal component was significant in the test for population differentiation and included it in downstream association analyses. The association between the genetic variants and prevalent or persistent hrHPV infection was estimated using unconditional multivariate logistic regression, assuming an additive genetic model adjusted for age, HIV status and the first principal component. Genome-wide significance was set at *p*-value < 5 × 10^− 8^. We used an additive genetic model adjusted for HIV status to test for replication of SNPs associated with HPV and cervical neoplasia in other populations and considered *p*-values < 0.05 as statistically significant evidence for replication. The analyses were conducted using PLINK.

## Results

The mean (±SD) age of the participants was 38 (±8) years while their mean (±SD) body mass index (BMI [kg/m^2^]) was 27 (±6). About half of the participants were HIV positive (52%, 270/517), 24% (125/517) had prevalent cervical hrHPV infections at baseline and 11% (51/473) had persistent hrHPV infections. The distribution of type-specific prevalent and persistent cervical hrHPV infections is shown in Table 1. Non HPV16/18 were more prevalent in the study population. The prevalence of HPV16 and HPV18 in the study population were 2% (10/517) and 4% (8/517), respectively. About 8% (10/125) of the women with cervical hrHPV infections had HPV16 and 16% (20/125) had HPV18 at baseline. HPV52 and HPV35 were the most prevalent HPV genotypes in the study population. About 7% (37/517) of the study population had HPV52, which accounted for about a third of the HPV positive infections at baseline. HPV52 and HPV35 were also more likely to persist, compared to the other hrHPV types. About 19% of the participants had single cervical hrHPV infections and ~ 9% of the participants had multiple cervical hrHPV infections at both visits. Participants returned for follow-up visits at a median (IQR) time of 5.7 (5.4–7.5) months.

**Table 1:** Distribution of Type-Specific Prevalent and Persistent Cervical High-Risk (hr) HPV Infections by HIV status

HPV Type	Prevalent Infections			Persistent Infections		
	Total *n* = 517	HIV Negative *n* = 247	HIV Positive *n* = 270	Total *n* = 473	HIV Negative *n* = 223	HIV Positive *n* = 250
hrHPV Positive	125 (24.2%)	37 (14.9%)	88 (32.6%)	51 (10.8%)	10 (0.0%)	41 (1.8%)
HPV 16	10 (1.9%)	2 (0.8%)	8 (2.9%)	4 (0.9%)	0 (0.0%)	4 (1.6%)
HPV 18	20 (3.9%)	5 (2.0%)	15 (5.5%)	5 (1.1%)	0 (0.0%)	5 (2.0%)
HPV 31	11 (2.1%)	1 (0.4%)	10 (3.7%)	6 (1.3%)	1 (0.5%)	5 (2.0%)
HPV 33	16 (3.1%)	3 (1.2%)	13 (4.8%)	4 (0.9%)	1 (0.5%)	3 (1.2%)
HPV 35	27 (5.2%)	5 (2.0%)	22 (8.1%)	14 (3.0%)	3 (1.4%)	11 (4.4%)
HPV 39	7 (1.3%)	2 (0.8%)	5 (1.8%)	2 (0.4%)	0 (0.0%)	2 (0.8%)
HPV 45	9 (1.7%)	4 (1.6%)	5 (1.8%)	1 (0.2%)	0 (0.0%)	1 (0.4%)
HPV 51	9 (1.7%)	1 (0.4%)	8 (2.9%)	3 (0.6%)	0 (0.0%)	3 (1.2%)
HPV 52	37 (7.1%)	12 (4.8%)	25 (9.2%)	18 (3.8%)	5 (2.2%)	13 (5.2%)
HPV 56	9 (1.7%)	4 (1.6%)	5 (1.8%)	1 (0.2%)	0 (0.0%)	1 (0.4%)
HPV 58	10 (1.9%)	2 (0.8%)	8 (2.9%)	3 (0.6%)	1 (0.5%)	2 (0.8%)
HPV 59	7 (1.3%)	0 (0.0%)	7 (2.5%)	0 (0.0%)	0 (0.0%)	0 (0.0%)

The table shows the number (percentage) of participants who were high-risk HPV positive at baseline

**Fig. 1:**
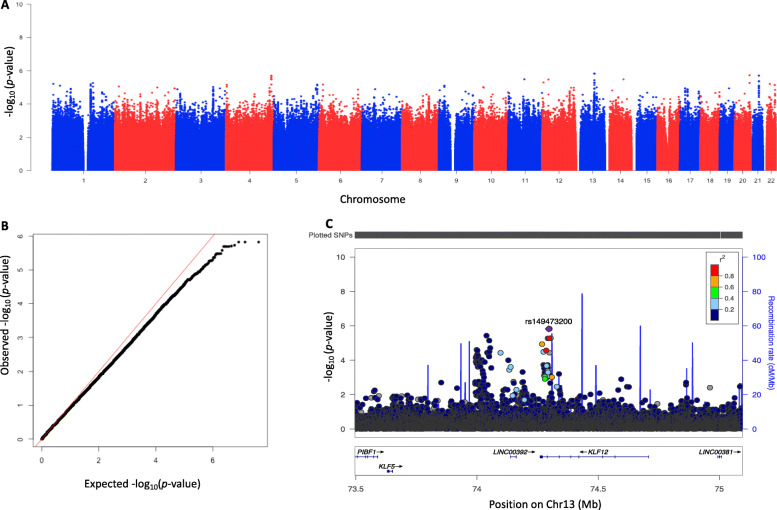
Genome-wide association results for prevalent high-risk HPV. λ = 1.02. **a** Manhattan plot (**b**) Quantile–quantile plot (**c**) Regional plot for rs149473200

The Manhattan plot, Fig. 1**,** shows all the SNPs and Table 2 shows the top 20 SNPs associated with prevalent cervical hrHPV infections. A cluster of SNPs (*D’* = 1, *r*^*2*^ = 1) located on chromosome 13, rs149473200, rs147344426 and rs151071053 (Odds Ratio [OR], *p*-value for all SNPs was OR: 7.06, *p* = 1.43 × 10^− 6^), had the strongest association with cervical prevalent hrHPV. The regional plot for rs149473200 in Fig. 1 shows that the cluster of SNPs are intronic in Krüppel-like Factor 12 gene (*KLF12)* and shows the surrounding markers. SNPs near Long Intergenic Non-Protein Coding RNA 290 gene (*NCRNA00290)* also had a borderline genome-wide significant association with prevalent hrHPV.

**Table 2:** Associations of the Top 20 SNPS with Prevalent Cervical high-risk Infections

SNP	Chr	Base Position	Near gene	Reference allele	MAF	OR	95% CI	*P*-value
rs149473200	13	74,295,767	*KLF12*	G	0.03	7.06	3.19–15.63	1.43 × 10^− 6^
rs147344426	13	74,298,464	*KLF12*	G	0.03	7.06	3.19–15.63	1.43 × 10^− 6^
rs151071053	13	74,299,405	*KLF12*	T	0.03	7.06	3.19–15.63	1.43 × 10^− 6^
rs572823632	13	74,299,732	*KLF12*^a^	GAA	0.03	7.06	3.19–15.63	1.43 × 10^− 6^
rs73010973	4	181,310,690	*NCRNA00290*	T	0.06	3.86	2.22–6.72	1.70 × 10^− 6^
rs73010975	4	181,310,704	*NCRNA00290*	T	0.06	3.86	2.22–6.72	1.70 × 10^− 6^
rs74739185	4	181,311,019	*NCRNA00290*	G	0.06	3.86	2.22–6.72	1.70 × 10^− 6^
rs79140020	4	181,311,020	*NCRNA00290*	T	0.06	3.86	2.22–6.72	1.70 × 10^− 6^
rs35833676	21	32,961,256	*TIAM1*	C	0.07	3.45	2.07–5.73	1.79 × 10^− 6^
rs3818252	20	58,675,675	*C20orf197*	C	0.57	2.22	1.60–3.10	2.06 × 10^− 6^
rs112893815	4	181,311,254	*NCRNA00290*	C	0.06	3.77	2.17–6.53	2.22 × 10^− 6^
–	8	74,725,256	*UBE2W*^a^	G	–	3.31	2.01–5.44	2.42 × 10^− 6^
rs506594	11	64,162,897	*RPS6KA4*	T	0.77	0.44	0.31–0.62	2.67 × 10^− 6^
rs73010952	4	181,308,382	*NCRNA00290*	G	0.06	3.69	2.14–6.38	2.77 × 10^− 6^
rs111800742	4	181,308,587	*NCRNA00290*	C	0.06	3.69	2.14–6.38	2.77 × 10^− 6^
rs73010957	4	181,309,080	*NCRNA00290*	A	0.06	3.69	2.14–6.38	2.77 × 10^− 6^
rs6574170	14	74,729,207	*VSX2*	A	0.04	5.29	2.63–10.67	3.16 × 10^− 6^
–	17	36,374,963	*LOC440434*^a^	TA	–	0.48	0.35–0.65	3.17 × 10^− 6^
rs17090215	13	74,039,057	*KLF12*	C	0.05	3.90	2.20–6.92	3.21 × 10^−6^
rs375435036	1	72,615,562	*NEGR1*^a^	C	0.15	2.52	1.71–3.73	3.49 × 10^− 6^

For this analysis, 125 women with cervical hrHPV infections were compared to 392 women without cervical hrHPV infections at baseline. Base positions were based on hg19; ^a^The variant is not in 1000 genomes v1, the nearest gene was obtained from variants surrounding the base location on the specific chromosome; Odds Ratio (OR) and 95% Confidence Intervals (CI) were estimated using an additive genetic model; Models were adjusted for age, HIV status and the first principal components of the genotypes

**Fig. 2:**
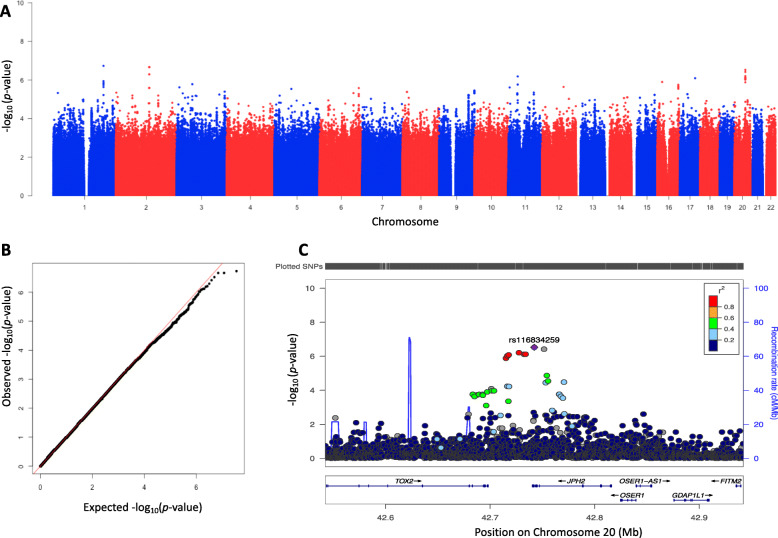
Genome-wide association results for persistent high-risk HPV. λ = 1.00. **a** Manhattan plot (**b**) Quantile–quantile plot (**c**) Regional plot for rs116834259

The SNP with the strongest association was located on Chr5:10847898, OR: 6.86, *p* = 7.15 × 10^− 8^, Table 3. This variant has not been included in the 1000 Genomes data resources. However, we found that the variants surrounding this region, Chr5:10847888–10,847,902, are located between Death Associated Protein gene (*DAP*) and Catenin Delta 2 (*CTNND2*) genes. Figure 2 shows a Manhattan plot and a regional plot of association with persistent cervical hrHPV infections. Other top variants associated with persistent hrHPV infections were rs200516199 upstream of MicroRNA 365b gene *(MIR365–2),* OR: 7.71, *p* = 2.63 × 10^− 7^; variants clustered upstream of Nuclear Receptor Subfamily 5 Group A Member 2 gene *(NR5A2)* and Junctophilin Type 2 gene *(JPH2)*. Next, we conducted a replication study by identifying all SNPs associated with HPV and cervical neoplasia in other studies (Supplementary Table 1) and evaluated their association with hrHPV in the present study, using an adjusted additive genetic model. We found rs9893818 (OR: 0.88, *p* = 0.58 for prevalent hrHPV; OR: 0.92, *p* = 0.82 for persistent hrHPV) and rs2299187 (OR: 0.95, *p* = 0.86 for prevalent hrHPV; OR: 1.13, *p* = 0.71 for persistent hrHPV) in our dataset but they were not significantly associated with prevalent or persistent cervical hrHPV infections (Supplemental Table 2). Lastly, we conducted stratified analysis by HIV status and found that none of the variants reached genome-wide statistical significance (Supplemental Tables 3 and 4).

**Table 3:** Associations of the Top 20 SNPS with Persistent Cervical high-risk Infections

SNP	Chr	Base Position	Near gene	Reference allele	MAF	OR	95% CI	*P*-value
–	5	10,847,898	*DAP*^a^	C	–	6.87	3.41–13.84	7.15 × 10^−8^
rs143668247	1	199,701,882	*NR5A2*	C	0.35	3.66	2.24–5.97	2.03 × 10^−7^
rs200516199	17	29,917,727	*MIR365–2*	TTTGA	0.04	7.71	3.54–16.78	2.63 × 10^− 7^
rs116834259	20	42,742,258	*JPH2*	T	0.11	4.42	2.50–7.81	3.15 × 10^− 7^
rs11452236	20	42,751,590	*JPH2*	C	0.13	4.03	2.34–6.92	4.78 × 10^− 7^
rs74358070	20	42,727,587	*JPH2*	T	0.11	4.24	2.41–7.47	5.52 × 10^−7^
rs16832308	2	133,253,243	*GPR390*	A	0.04	6.30	3.05–13.01	6.49 × 10^−7^
rs62167448	2	133,254,091	*GPR39*	C	0.04	6.30	3.05–13.01	6.49 × 10^−7^
rs150410476	11	37,223,676	*C11orf74*	A	0.03	8.31	3.60–19.16	6.80 × 10^−7^
rs12740341	1	199,699,781	*NR5A2*	C	0.26	3.18	2.01–5.03	7.94 × 10^−7^
rs79032354	20	42,732,460	*JPH2*	C	0.13	3.91	2.27–6.71	7.96 × 10^−7^
rs6130527	20	42,733,946	*JPH2*	G	0.13	3.91	2.27–6.71	7.96 × 10^−7^
rs4810411	20	42,751,472	*JPH2*	C	0.13	3.81	2.24–6.50	7.99 × 10^−7^
rs2502139	1	199,695,633	*NR5A2*	A	0.27	3.17	2.00–5.02	8.94 × 10^−7^
rs34026413	1	199,695,074	*NR5A2*	A	0.27	3.10	1.97–4.88	7.94 × 10^−6^
–	1	26,304,144	*PAFAH2*^a^	C	–	10.19	4.01–25.92	7.94 × 10^− 6^
rs543084794	2	133,250,145	*GPR39*^a^	C	0.04	6.44	3.04–13.65	7.94 × 10^− 6^
rs79014529	20	42,717,781	*TOX2*	C	0.13	3.81	2.22–6.53	7.94 × 10^− 6^
rs1429702	1	199,694,059	*NR5A2*	T	0.27	3.10	1.96–4.87	7.94 × 10^−6^
rs6130520	20	42,716,399	*TOX2*	G	0.13	3.77	2.20–6.47	7.94 × 10^−6^

For this analysis, 51 women with hrHPV infection at both the baseline and follow-up visits were compared to 355 women without hrHPV infections at either the baseline or follow-up visits. Base positions were based on hg19; ^a^The variant is not in 1000 genomes v1, the nearest gene was obtained from variants surrounding the base location on the specific chromosome; Odds Ratio (OR) and 95% Confidence Intervals (CI) were estimated using an additive genetic model; Models were adjusted for age, HIV status and the first principal components of the genotypes

## Discussion

This is the first GWAS of cervical hrHPV infection, to our knowledge. The top three variants associated with prevalent cervical hrHPV infection were clustered in *KLF12*, while those associated with persistent cervical hrHPV infection were near *DAP, CTNND2, MIR365–2 and NR5A2*. These associations were borderline genome-wide significant. It is well established that the determinants of prevalent and persistent cervical hrHPV infections are different. Our finding of separate variants associated with prevalent and persistent cervical hrHPV suggests that their genetic risk factors may also differ.

The associated SNPs in *KLF12,* rs149473200 and rs147344426, are eQTLs of CD3e molecule (*CD3E*), a protein coding gene which plays an essential role in T-cell development and its defects cause immunodeficiency. *KLF12*, a protein coding gene, is overexpressed in human B and T lymphocytes, CD8 T cells and natural killer cells [25]. These cells play important roles during immune response to hrHPV infection, including recognizing and destroying infected cells. hrHPV causes the immune system to become more tolerant to infection by avoiding cytolysis of host cells, inhibiting interferon synthesis and cytotoxic T cell function, and inducing regulatory T cell infiltration [26–28]. This creates a cervical microenvironment that is susceptible to persistent infection and carcinogenesis. *KLF12* has been linked to several cancers [29–32], including head and neck cancers [33, 34], which are usually associated with hrHPV. A study of HPV integration breakpoints in the human genome showed that a copy of the virus was integrated between *KLF5* and *KLF12* in HPV-positive SiHa cells [35]. Recently, a whole-genome sequencing study on HPV-positive SiHa, HeLa and cervical carcinoma cells showed *KLF12* was one of the top three integration sites for HPV [36]. Thus, *KLF12* may play a major role in the underlying mechanisms that lead to hrHPV infection, persistence and cervical carcinogenesis.

A locus between *CTNND2 and DAP* in the short arm of chromosome 5*,* had the strongest association with persistent cervical hrHPV infections in the present study. *CTNND2* gene encodes an adhesive junction associated protein and is overexpressed in the cervix [25]. It has been implicated in cancer formation and has been linked to breast and ovarian cancers [37–39]. *DAP* encodes a basic, proline-rich protein which acts as a positive mediator of programmed cell death that is induced by interferon-gamma [40]. It negatively regulates autophagy and is a substrate for mammalian target of rapamycin (mTOR) [41], which regulates different cellular processes. Results from GWASs show that *DAP* is associated with digestive disorders, gut microbiota, height and obesity [42–45]. There is some evidence that this gene plays a pro-apoptotic role in breast and cervical cancers [46–48]. Esteller *et. al.* showed that hypermethylation of the CpG islands located in the promoter region of *DAP* leads to transcriptional silencing thereby enabling malignant growth [49].

rs200516199 and rs143668247 near *MIR365–2 and NR5A2* (*LRH-1*), respectively, were also associated with persistent cervical hrHPV infections. Like *CTNND2* and *DAP*, *MIR365–2* has also been linked to breast and cervical cancers [50, 51]. It appears to have an oncogenic effect in some cancers [52, 53] and tumor suppressor effect in others [54–57]. Bioinformatics and experimental research studies have proved that apoptotic markers BAX and BCL-2, are two of the main targets of this microRNA [58, 59]. rs143668247 alters motifs in POU Class 5 Homeobox 1 (*POU5F1*) gene. Aberrant expression of this gene in adult tissues is associated with tumorigenesis [37]. rs143668247 is located 295 kb 5′ of *NR5A2,* an orphan receptor recently identified as a negative modulator of hepatic inflammatory processes [60]. It encodes a protein which is highly expressed in the liver and is involved in regulating the expression of genes for lipid metabolism, hepatitis B virus [61, 62] and several cancers [63–69]. Although these genes have not been previously linked to HPV infection, subsequent GWAS may confirm our findings.

Our study is limited by its exploratory nature. Given the small sample size of this study the power of the study was limited. Thus, we may have missed associations with smaller effect sizes and we could not examine the relationship between variants and type-specific hrHPV and by HIV status. Our replication study yielded two SNPs, *TMC6/TMC8*:rs9893818 which was reported to be associated with CIN3/cervical cancer [9] and *CACNA2D1*:rs2299187, which was associated with survival of head and neck squamous cell carcinoma in a recent GWAS [70]. However, these variants were not associated with hrHPV in our study. Also, rs7082598 variant in *PRDX3* and rs2305809 variant in *RPS19*, which were shown to be associated with HPV persistence in a candidate gene study conducted within Guanacaste cohort in Costa Rica, were not associated with hrHPV in our study. This may be due to inadequate sample size, variability in the types of hrHPV or population differences. Unlike our study population which was comprised of only African women, the population of Guanacaste is heavily admixed and has been described as being composed mainly of European (42.5%) and Native American (38.3%) ancestries, with considerable African influence (15.2%) and a small influence from Asians (4%) [71]. The frequency of rs7082598 is 0.14 (AFR), 0.11 (AMR), 0.04 (ASN) and 0.08 (EUR) [72], our study may have been underpowered to detect an association with this variant. The frequency of rs2305809 is 0.89 (AFR), 0.52 (AMR), 0.56 (ASN) and 0.48 (EUR) [72], suggesting that most African women have this variant regardless of their HPV status, which is most likely why we were unable to detect an association between rs2305809 and HPV in our study population. The findings from this exploratory study suggests that there are significant associations between genetic variants and cervical hrHPV infection and larger studies are warranted. The strengths of our study include studying a well-characterized longitudinal cohort with multiple hrHPV assessments in the participants. Secondly, the main loci identified have biological and functional support for a role in HPV infection and persistence. Lastly, the variant frequencies we observed were similar between our samples and those of west African ancestry samples in the 1000 Genomes dataset, validating the genotype accuracy in our datasets.

## Conclusion

In conclusion, our study yielded suggestive genetic risk factors for prevalent and persistent cervical hrHPV infections. Further investigations of genetic variation in the *KLF12, CTNND2* and *DAP* genes may provide insight into mechanisms of susceptibility to hrHPV infection and persistence. Larger discovery and replication studies are warranted to confirm these findings.

## Supplementary Information

**Additional file 1: Supplemental Table S1**.

**Additional file 2: Supplemental Table S2**. Replication of SNPs Associated with Cervical high-risk Infections.

**Additional file 3: Supplemental Table S3**. Associations of the Top SNPS with Cervical High-risk Infections in HIV-Negative Women.

**Additional file 4: Supplemental Table S4**. Associations of the Top SNPS with Cervical High-risk Infections in HIV-Positive Women.

**Additional file 5: Supplementary Figure S1**. Principal components (PC) plot of the genotypes of the study participants.

## Data Availability

The datasets used and/or analyzed during the current study are available from the corresponding author on reasonable request.

## Abbreviations

AGVP: African Genome Variation Project
BMI: Body mass index
CIN: Cervical intraepithelial neoplasia
DEIA: DNA Enzyme Immunoassay
GWAS: Genome-wide association studies
HPV: Human papillomavirus
hrHPV: High-risk Human Papillomavirus
HWE: Hardy-Weinberg equilibrium
IQR: Interquartile range
MAF: Minor allele frequency
MEGA: Multi-Ethnic Global Array
OR: Odds ratio
PBWT: Positional Burrows-Wheeler transform
SNP: Single nucleotide polymorphisms
UG2G: Uganda 2000 Genomes Project
