# Supplementary material for: Genome-wide association study of prevalent and persistent cervical high-risk human papillomavirus (HPV) infection
Source: BMC Med Genet. 2020 Nov 23;21:231. doi: 10.1186/s12881-020-01156-1 (PMC7682060; doi:10.1186/s12881-020-01156-1)
Supplement: Supplementary file 2 — Additional file 2: Supplemental Table S2. Replication of SNPs Associated with Cervical high-risk Infections. [file 12881_2020_1156_MOESM2_ESM.docx]

| **Supplemental Table 2.** Replication of SNPs Associated with Cervical high-risk Infections | | | | | | | | | |
| --- | --- | --- | --- | --- | --- | --- | --- | --- | --- |
| Study Population | Disease/Trait | SNP | Chr | Base Position | Near gene | Ref allele | MAF | OR | P-value |
| Guancaste | CIN3/CaCervix | rs9893818 | 17 | 76144167 | *TMC6/8* | - | - | - | 0.0081 |
| Present Study | HPV Prevalence | rs9893818 | 17 | 76144167 | *TMC6/8* | C | 0.14 | 0.88 | 0.58 |
| Present Study | HPV Persistence | rs9893818 | 17 | 76144167 | *TMC6/8* | C | 0.14 | 4.00 | 0.82 |
|  |  |  |  |  |  |  |  |  |  |
| Mainly EUR | Ca Head and Neck | rs2299187 | 7 | 82047102 | *CACNA2D1* | - | - | 3.26 | 8 x 10^-6^ |
| Present Study | HPV Prevalence | rs2299187 | 7 | 82047102 | *CACNA2D1* | C | 0.08 | 0.92 | 0.82 |
| Present Study | HPV Persistence | rs2299187 | 7 | 82047102 | *CACNA2D1* | C | 0.08 | 1.14 | 0.71 |

Ca = Cancer; Ref = Reference
