# Supplementary material for: Genome-wide association study of prevalent and persistent cervical high-risk human papillomavirus (HPV) infection
Source: BMC Med Genet. 2020 Nov 23;21:231. doi: 10.1186/s12881-020-01156-1 (PMC7682060; doi:10.1186/s12881-020-01156-1)
Supplement: Supplementary file 3 — Additional file 3: Supplemental Table S3. Associations of the Top SNPS with Cervical High-risk Infections in HIV-Negative Women. [file 12881_2020_1156_MOESM3_ESM.docx]

| **Supplemental Table 3.** Associations of the Top SNPS with Cervical High-risk Infections in HIV-Negative Women | | | | | | | | |
| --- | --- | --- | --- | --- | --- | --- | --- | --- |
| SNP | Chr | Base Position | Near gene | Reference allele | MAF | | OR | P-value |
| Prevalent hrHPV | |  |  |  |  | |  |  |
| rs111210036 | 16 | 86142078 | *KLF12* | T | - | 5.92 | | 3.35 X 10^-7^ |
| rs111210034 | 16 | 86141779 | *KLF12* | A | - | 4.86 | | 2.94 X 10^-6^ |
| rs79725749 | 5 | 10117667 | *CTD-2199O4.1* | G | 0.07 | 5.97 | | 3.30 X 10^-6^ |
| rs79443954 | 5 | 10117702 | *CTD-2199O4.1* | C | 0.07 | 5.97 | | 3.30 X 10^-6^ |
| rs200837604 | 9 | 96151676 | *U6* | G | 0.04 | 23.80 | | 4.45 X 10^-6^ |
| rs190396612 | 11 | 122480343 | *U6* | G | 0.04 | 8.86 | | 4.75 X 10^-6^ |
| rs141342775 | 4 | 168901531 | *RP11-310I9.1* | C | 0.06 | 21.73 | | 5.48 X 10^-6^ |
| rs73864809 | 4 | 168908513 | *RP11-310I9.1* | C | 0.07 | 21.73 | | 5.48 X 10^-6^ |
| rs185123437 | 8 | 28549275 | *EXTL3* | G | 0.13 | 6.58 | | 6.33 X 10^-6^ |
| rs73864815 | 4 | 168952026 | *RP11-310I9.1* | T | 0.07 | 20.79 | | 7.10 X 10^-6^ |
| Persistent hrHPV | |  |  |  |  | |  |  |
| rs116031905 | 12 | 77813516 | *RP1-34H18.1* | G | 0.04 | | 22.50 | 5.10 X 10^-5^ |
| - | 17 | 37317767 | *-* | G | - | | 51.54 | 5.15 X 10^-5^ |
| rs8007107 | 14 | 61629275 | *PRKCH* | T | 0.16 | | 8.84 | 9.51 X 10^-5^ |
| rs113019802 | 2 | 46884824 | *CRIPT* | G | 0.04 | | 155.83 | 1.13 X 10^-4^ |
| rs28497064 | 1 | 16917877 | *NBPF1* | A | - | | 17.35 | 1.24 X 10^-4^ |
| rs10827564 | 10 | 36222623 | *RP11-309N24.1* | G | 0.04 | | 50.09 | 1.53 X 10^-4^ |
| rs62228385 | 22 | 44929741 | *LINC00207* | C | 0.05 | | 41.24 | 1.57 X 10^-4^ |
| rs41524545 | 11 | 35585540 | *RP5-945I17.2* | T | 0.22 | | 13.53 | 1.70 X 10^-4^ |
| rs60098438 | 11 | 35587723 | *RP5-945I17.2* | C | 0.22 | | 13.53 | 1.70 X 10^-4^ |
| rs60618536 | 11 | 35588097 | *RP5-945I17.2* | C | 0.22 | | 13.53 | 1.70 X 10^-4^ |
