# Supplementary material for: Genome-wide association study of prevalent and persistent cervical high-risk human papillomavirus (HPV) infection
Source: BMC Med Genet. 2020 Nov 23;21:231. doi: 10.1186/s12881-020-01156-1 (PMC7682060; doi:10.1186/s12881-020-01156-1)
Supplement: Supplementary file 4 — Additional file 4: Supplemental Table S4. Associations of the Top SNPS with Cervical High-risk Infections in HIV-Positive Women. [file 12881_2020_1156_MOESM4_ESM.docx]

| **Supplemental Table 4.** Associations of the Top SNPS with Cervical High-risk Infections in HIV-Positive Women | | | | | | | | |
| --- | --- | --- | --- | --- | --- | --- | --- | --- |
| SNP | Chr | Base Position | Near gene | Reference allele | MAF | | OR | P-value |
| Prevalent hrHPV | |  |  |  |  | |  |  |
| rs56828769 | 3 | 174165396 | *NAALADL2* | A | 0.08 | 6.99 | | 1.36 X 10^-6^ |
| rs146409073 | 3 | 174243353 | *NAALADL2* | A | 0.08 | 8.56 | | 1.39 X 10^-6^ |
| rs142162751 | 11 | 59963023 | *MS4A4E* | A | 0.06 | 6.19 | | 2.99 X 10^-6^ |
| rs186320956 | 11 | 59965073 | *MS4A4E* | T | 0.06 | 6.19 | | 2.99 X 10^-6^ |
| rs10080423 | 6 | 155013345 | *SCAF8* | T | 0.45 | 2.54 | | 3.72 X 10^-6^ |
| rs3844135 | 11 | 60025356 | *MS4A4E* | G | 0.09 | 5.02 | | 4.27 X 10^-6^ |
| rs34136369 | 10 | 36183261 | *RP11-309N24.1* | CAA | 0.14 | 3.92 | | 4.48 X 10^-6^ |
| rs11230188 | 11 | 59971661 | *MS4A4E* | T | 0.08 | 5.73 | | 4.71 X 10^-6^ |
| rs9938998 | 16 | 58889816 | *RP11-700H13.1* | T | 0.15 | 3.98 | | 5.13 X 10^-6^ |
| rs114201684 | 11 | 59928625 | *AP001257.1* | A | 0.07 | 4.92 | | 5.52 X 10^-6^ |
| Persistent hrHPV | |  |  |  |  | |  |  |
| - | 5 | 10847898 | *-* | C | - | | 8.71 | 2.74 X 10^-7^ |
| rs11452236 | 20 | 42751590 | *JPH2* | C | 0.12 | | 5.43 | 3.08 X 10^-7^ |
| - | 8 | 9379246 | *-* | C | - | | 6.78 | 3.28 X 10^-7^ |
| - | 20 | 42751472 | *-* | C | - | | 4.98 | 5.52 X 10^-7^ |
| rs116834259 | 20 | 42742258 | *JPH2* | T | 0.09 | | 4.99 | 9.29 X 10^-7^ |
| rs7268343 | 20 | 42754240 | *JPH2* | G | 0.13 | | 5.23 | 9.85 X 10^-7^ |
| rs115395357 | 11 | 37218328 | *SNORA31* | A | 0.04 | | 14.48 | 1.24 X 10^-6^ |
| rs79032354 | 20 | 42732460 | *JPH2* | C | 0.09 | | 4.51 | 1.28 X 10^-6^ |
| rs6130527 | 20 | 42733946 | *JPH2* | G | 0.09 | | 4.51 | 1.28 X 10^-6^ |
| rs74358070 | 20 | 42727587 | *JPH2* | T | 0.09 | | 4.85 | 1.29 X 10^-6^ |
