# Supplementary material for: Genome-wide association study of prevalent and persistent cervical high-risk human papillomavirus (HPV) infection
Source: BMC Med Genet. 2020 Nov 23;21:231. doi: 10.1186/s12881-020-01156-1 (PMC7682060; doi:10.1186/s12881-020-01156-1)

**Supplementary Figure**: Principal components (PC) plot of the genotypes of the study participants


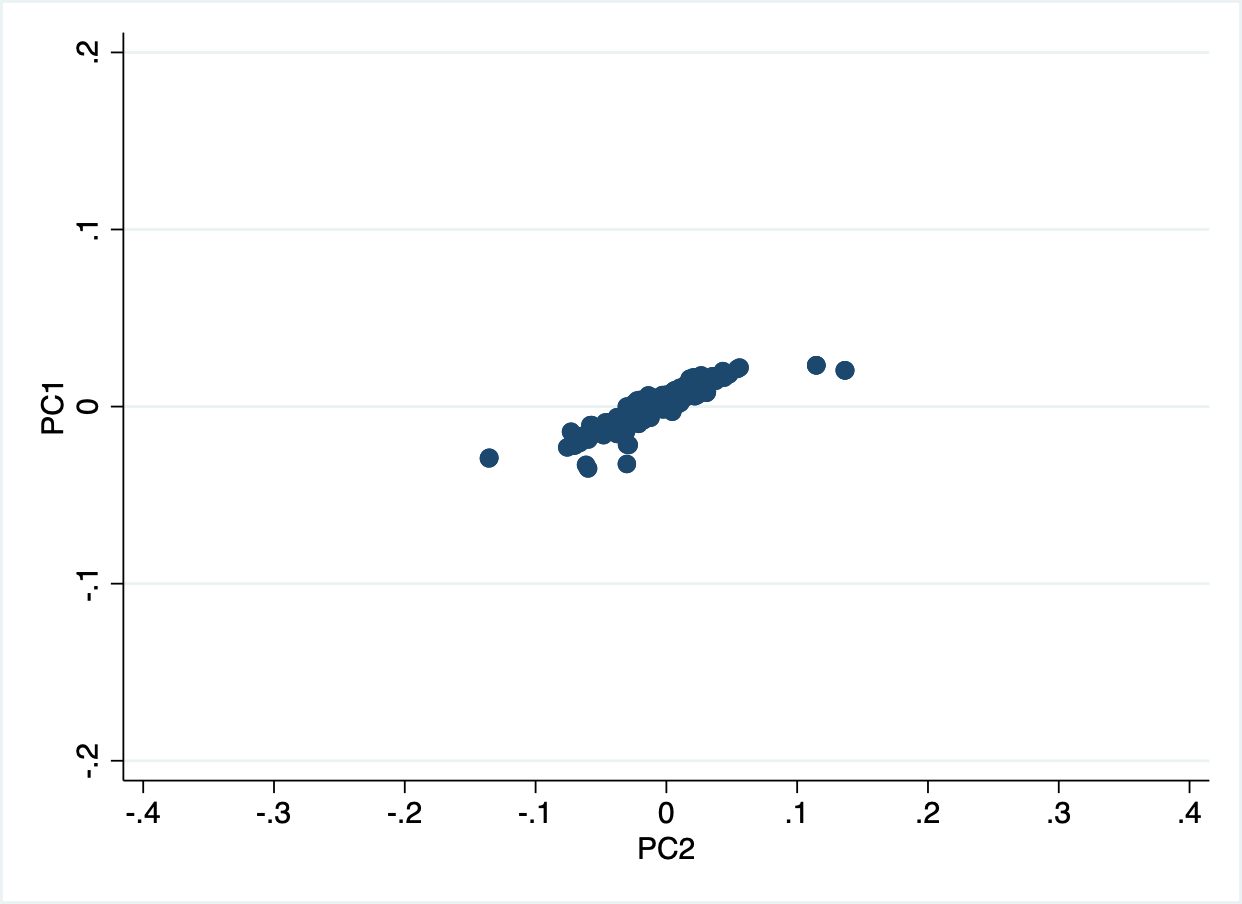

Supplement: Supplementary file 5 — Additional file 5: Supplementary Figure S1. Principal components (PC) plot of the genotypes of the study participants. [file 12881_2020_1156_MOESM5_ESM.docx]
